# The Spatial Distribution and Genetic Diversity of the Soybean Cyst Nematode, *Heterodera glycines*, in China: It Is Time to Take Measures to Control Soybean Cyst Nematode

**DOI:** 10.3389/fpls.2022.927773

**Published:** 2022-06-15

**Authors:** Yun Lian, Georg Koch, Dexin Bo, Jinshe Wang, Henry T. Nguyen, Chun Li, Weiguo Lu

**Affiliations:** ^1^Henan Academy of Crops Molecular Breeding, National Centre for Plant Breeding, Zhengzhou Subcenter of National Soybean Improvement Center, Key Laboratory of Oil Crops in Huang Huaihai Plains, Ministry of Agriculture and Rural Affairs, Henan Provincial Key Laboratory for Oil Crops Improvement, Zhengzhou, China; ^2^National Centre for Plant Breeding, Xinxiang, China; ^3^Hubei Key Laboratory of Agricultural Bioinformatics, Huazhong Agricultural University, Wuhan, China; ^4^Division of Plant Sciences, University of Missouri, Columbia, MO, United States

**Keywords:** soybean, soybean cyst nematode (*Heterodera glycines*) (SCN), China, virulence, resistance, re-sequencing, sustainable agriculture

## Abstract

The continuous evolution and spread of virulent forms of the soybean cyst nematode (SCN) driven by the environment and anthropogenic intervention is a serious threat to the soybean production worldwide, including China. Especially in China, the implemented measures to control SCN are insufficient for sustainable agricultural development yet. We summarized our knowledge about the spread and spatial distribution of SCN in China and the virulence diversity in the main soybean growing areas. To reveal the genetic relatedness and diversity of SCN populations, we re-sequenced 53 SCN genomes from the Huang-Huai Valleys, one of the two main soybean growing areas in China. We identified spreading patterns linked to the local agroecosystems and topographies. Moreover, we disclosed the first evidence for the selection of complex virulence in the field even under low selection pressure in an example from North Shanxi. SCN is present in all soybean growing areas in China but SCN susceptible cultivars are still largely grown indicating that SCN-related damage and financial loss have not received the attention they deserve yet. To prevent increasing yield losses and to improve the acceptance of resistant cultivars by the growers, we emphasized that it is time to accelerate SCN resistance breeding, planting resistant cultivars to a larger extent, and to support farmers to implement a wider crop rotation for sustainable development of the soybean production in China.

## Introduction

*Heterodera glycines* (*H. glycines*, HG; soybean cyst nematode, SCN) is considered to be one of the most damaging pests in soybean worldwide, and the loss of revenue from SCN damage is estimated to be billions of dollars per year worldwide (Koenning and Wrather, 2010; Bradley et al., 2021). There are few sporadic reports that SCN is also widely distributed in China but not many actions taken to control it yet (Lian et al., 2016; Chen et al., 2021; Peng et al., 2021). Furthermore, it has also been reported that virulent nematode populations have developed over time and overcome the resistance of most known plant resistant sources, such as PI 88788 (Niblack et al., 2008; Mitchum, 2016). In some soybean planting areas, the dominant SCN populations appear to be shifting in their virulence composition and complexity (Lian et al., 2016; Howland et al., 2018), and an increasing number of HG types of the SCN (Niblack et al., 2002) were detected (Hua et al., 2018; Chen et al., 2021). Referring to the efficient mechanisms of the pathogen to overcome plant resistance genes and to minimize the accumulation of virulence genes, an efficient, proactive resistance gene management is needed to control the SCN damage in the soybean production (Gardner et al., 2017; Shaibu et al., 2020). This is particularly important with regard to China’s ‘Soybean Revitalization Plan’ launched in 2019, in which the goals are expressed to expand the soybean planting area (mostly used for food production), to improve crop yield and quality, and to promote the overall sustainable development of the soybean production. Reliable management of SCN control should be urgently implemented and assigned with increasing importance.

In this study, (1) we reviewed and summarized our knowledge about the overall spatial distribution of SCN in China for the first time to understand and assess the threat that the SCN exposes to the soybean production in China, (2) regarding the appearance of new, highly virulent races, we proposed to add two further indicator lines including ZDD 2315 and PI 567516C to the soybean host differential set proposed by Niblack et al. (2002) to enhance the resolving power for SCN virulence monitoring, (3) we analyzed the re-sequencing data of 53 SCN populations from the Huang-Huai Valleys, which provided new perspectives on the genomic diversity among SCN populations, the genetic relatedness of the geographic distribution, and the virulence complexity, and (4) we provided the first evidence for virulence selection even under low resistance pressure in North Shanxi and the Huang-Huai Valleys, and finally, we discussed systematic approaches for the management of SCN control.

### The Spatial Distribution of SCNs in China

**FIGURE 1:**
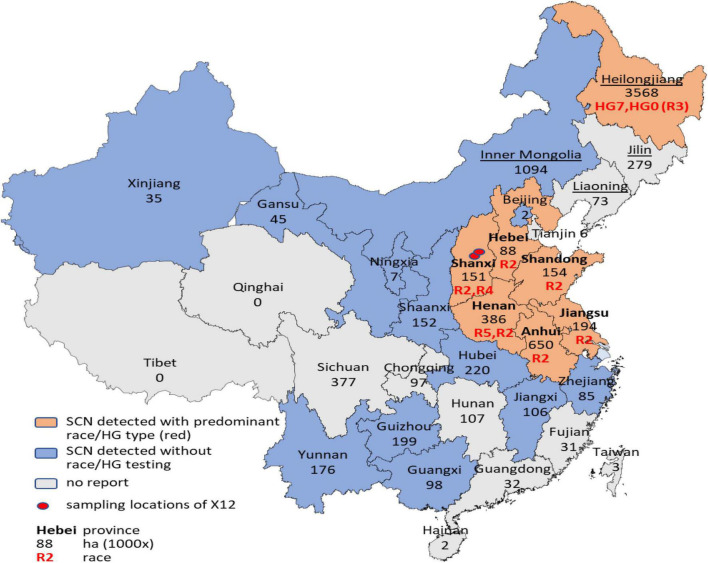
The spatial distribution of soybean cyst nematodes (SCNs) in China. The names of the provinces of the main growing area of the Huang-Huai Valleys are typed in bold, and the names of the provinces of the Northeast growing area are underlined. Black numbers in the figure represent the soybean growing area in thousands of hectares (1,000 × ha) of 2018 (China Agriculture Yearbook). Red text indicates the predominant race(s) or HG type(s).

The two major soybean growing areas in China are the Huang-Huai Valleys in Central-East China and Northeast China with a combined soybean planting area exceeding 50% of the national planting area for decades and occupying 78.9% according to the latest official statistics in 2018 (Figure 1). The predominant SCN races in these two major soybean growing areas in China were race 2, HG types 1.2.5.6.7-/1.2.5. 7-, in the Huang-Huai Valleys, surveyed in 2012–2015 (Lian et al., 2016), and race 3, HG Type 7-, in Northeast (Heilongjiang Province), surveyed in 2015 (Hua et al., 2018; Chen et al., 2021). Recent reports (Zheng et al., 2009; Peng et al., 2015) have shown that SCN has further spread into minor soybean planting areas where SCN was not detected before, such as Xinjiang Uygur Autonomous Region, Guangxi, and Guizhou Province. Despite the ubiquitous occurrence of SCN, resistant cultivars are almost not available or only recently registered but not widely used in China. But unfortunately, resistance against SCN is not included in the Chinese variety registration system. To find out which varieties are resistant to SCN, 170 cultivars and breeding lines for the use in the Huang-Huai Valleys were screened with Kompetitive allele-specific PCR (KASP) markers specific for the most common SCN resistance genes *Rhg1* and *Rhg4* (Kadam et al., 2016) and have revealed that only one cultivar and five breeding lines harbor SCN resistance loci (Lian et al., 2019a). Of these, only the cultivar “Shangdou1201” (Peking-type resistance), registered in 2020, is commercially grown but on a very limited scale and accounting for only 0.6% of the Huang-Huai Valleys soybean planting area in 2020. In addition, we tested the three cultivars with the largest acreage in Heilongjiang and Huang-Huai Valleys in 2019 with another set of KASP markers for the same SCN-resistant genes (Shi et al., 2015). The results showed that only one variety, named “Heinong 84,” showed SCN resistance (Peking-type resistance). This cultivar, registered in 2017, was grown on only 4% of Heilongjiang’s soybean planting area in 2019, while none of the three cultivars with the largest acreage in Huang-Huai Valleys showed SCN resistance.

Regarding the marginal cultivation of resistant cultivars, it is puzzling that virulent forms of SCN have evolved (Lian et al., 2017; Hua et al., 2018) and the predominate races have shifted over time to more virulent races (Lian et al., 2016; Chen et al., 2021). Furthermore, a new, highly virulent population, named X12, appeared (Lian et al., 2017), which is virulent on all the four indicator lines of the race test (Riggs and Schmitt, 1988), all the seven indicator lines of the HG type test (Niblack et al., 2002), and also the resistant germplasm of PI 567516C collected from Shandong Province, China (Diers et al., 1997; Chen et al., 2006) (female index = 121, unpublished own data). It is also virulent on ZDD 2315, the most promising elite germplasm developed in China, which is resistant to all SCN populations identified so far except for X12 (Lian et al., 2017). In Figure 1, the spatial distribution of SCN overall soybean growing areas in China is summarized for the first time.

### Proposal of ZDD 2315 and PI 567516C as Additional Differentials to Differentiate Newly Observed and Highly Virulent SCN Populations

The virulence of SCN populations is determined with various differential sets of indicator lines harboring different, known SCN resistant genes so far. Riggs and Schmitt (1988) proposed to describe the virulence of SCN populations based on four soybean indicator lines including Pickett, Peking, PI 88788, and PI 90763. Niblack et al. (2002) expanded the differential set and proposed seven soybean indicator lines including Peking, PI 88788, PI 90763, PI 437654, PI 89772, PI 209332, and PI 548316 and named the populations according to a new HG type classification considering the individual host/pathogen interaction. However, with the appearance of the highly virulent SCN populations LY1 and X12 (LY1 is a synthetic inbred population from crossing race 2 female with race 5 male (Arelli et al., 2009)), neither the race typing nor the HG type classification is able to differentiate race 4, LY1 and X12, essentially due to the lack of corresponding resistant differential lines. Further development of the host differential set is needed to differentiate these highly virulent SCN populations. Therefore, we proposed to add the elite line ZDD 2315, a Peking resistant germplasm with resistance against race 1 to race 5 identified in the 1980s by the Coordinative Group of Evaluation (CGE) of SCN organized by Chinese scientists (CGE, 1993) and the plant genetic resource PI 567516C to the differential set proposed by Niblack et al. (2002) to be able to differentiate the highly virulent SCN populations of race 4, X12 and LY1 (Table 1 and Supplementary Table 1).

**TABLE 1:** Differential reaction of the new, highly virulent X12 and LY1 populations but also race 4 on the newly proposed differential lines, ZDD 2315 and PI 567516C.

	Indicator plant line	
SCN population	ZDD 2315	PI 567516C
Race 4	–	+
X12	+	+
LY1	NT	–*

NT, not tested. *Published by Arelli et al. (2009).

The host/pathogen interaction is characterized as compatible, “ **+** ” (SCN is virulent), if the relative, observed female number is ≥ 10% of the corresponding female number on the susceptible control, usually cultivar Lee. “**–**” corresponds to an incompatible interaction (avirulence), if the relative, observed female number is <10% of the susceptible control.

### Genetic Diversity Analysis of 54 SCN Populations From the Huang-Huai Valleys

**FIGURE 2:**
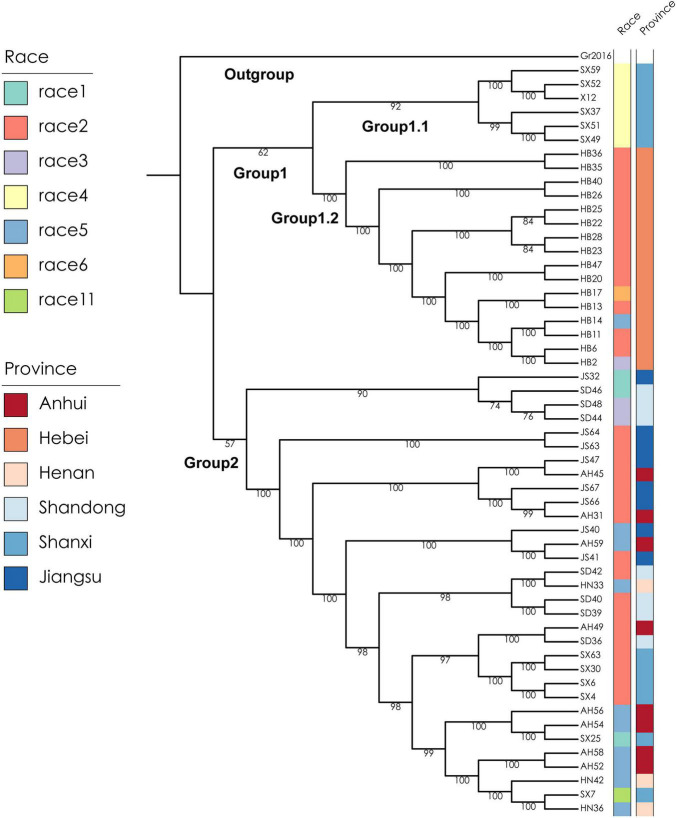
Maximum-likelihood (ML) phylogenetic tree of 54 populations of the SCN from the Huang-Huai Valleys in China. The two capital letters represent the abbreviations of the provinces where the soil samples were collected, followed by the sample number. AH, JS, SD, HN, HB, and SX refer to Anhui, Jiangsu, Shandong, Henan, Hebei, and Shanxi provinces, respectively. The left color bar on the right is showing the corresponding race number and the right color bar the collection province. *Globodera rostochiensis*, Gr2016, is used as outgroup species (Akker et al., 2016).

**FIGURE 3:**
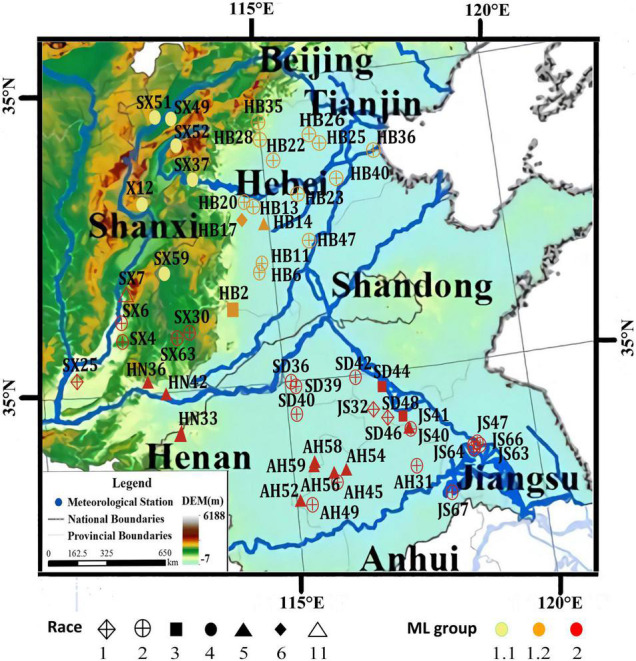
The spatial distribution of 54 populations of the SCN in the Huang-Huai Valleys in China. Adopted from Yuan et al. (2019) with permission. SCN sample location, race, and ML group added.

To better understand the genetic diversity of the SCN and the genetic relationship between spatially distributed populations, a subsample of 53 SCN populations from a former sampling survey (Lian et al., 2016) in the Huang-Huai Valleys comprising all race types and provinces of origin was selected and re-sequenced (Supplementary Table 2). X12 sequence was used as a reference genome (Lian et al., 2019b) and data of X12, a population originally from North Shanxi, were integrated, resulting in a total sample size of 54 populations. *Globodera rostochiensis*, Gr2016, was used as outgroup species (Akker et al., 2016). A total of 774,568 high-quality single nucleotide polymorphisms (SNPs) could be identified among the 54 SCN populations (Supplementary Table 2). A maximum-likelihood (ML) phylogenetic tree was constructed, and the 54 populations were clustered mainly into two groups (Figure 2). All 16 soil samples collected from Hebei Province (mostly identified as race 2) and the 6 soil samples collected from the northern part of Shanxi Province (including all the 5 samples identified as race 4 and 1 sample identified as X12) were distinctly clustered into a separate group designated as Group 1. The other 32 soil samples collected from Henan, Shandong, the southern part of Shanxi Province, Anhui, and Jiangsu Province (including the other 17 samples identified as race 2) were clustered into another, a more diverse separate group designated as Group 2. The samples of ML Group 1 were collected in the North of the Huang-Huai Valleys (Group 1.1, Group 1.2, Figure 3), whereas the samples of ML Group 2 were collected in the southern part of the Huang-Huai Valleys. These marked differences in virulence diversity, distribution, and complexity, i.e., all the highly virulent race 4 populations are included in Group 1.1 and all Hebei populations clustered in Group 1.2 and were mostly race 2, whereas in the South of the Huang-Huai Valleys, a higher diversity in virulence factors and genetic diversity is maintained, pointing to substantial different evolutionary factors active in the different growing areas. One of the reasons for the higher uniformity of SCN in Hebei may be that the cultivation area is topographically more uniform and spatially better interconnected than all other provinces; there are bordering mountains in the north and west^1^ and periodic sandstorms allowing intense wind spreading of soil^2^ and consequently also cysts, which additionally increases the risk of SCN spreading and increase of virulence complexity (and fitness). Furthermore, the North of the Huang-Huai Valleys (Hebei) marks the border area with a single-crop system in the North (1 crop per 12 months) and a double-crop system (2 crops within 12 months) in the South. Typically, the double-crop system involves a crop rotation, which is reducing selection pressure on pathogens explaining the lower virulence complexity in the South. The soil sample from the northern part of Shanxi Province (ML Group 1.1) represents a surprisingly high SCN virulence uniformity. It includes all the soil samples identified as containing race 4 SCN in this study (in the original more comprehensive surveys, only one additional race 4 sample was found in Hebei out of 112 SCN positive soil samples in total (Lian et al., 2016)). Race 4 is the most complex race with virulence against all 4 resistance hosts in the race differential set. The southern part of Huang-Huai Valleys, Group 2, showed more different virulence patterns and less virulence factors per population, indicating contrariwise strong selection pressure in the geographically diverse, hilly, and small-scaled structured cropping areas in North Shanxi, Group 1.1. Regarding the high virulence complexity of race 4, it may be not surprising that X12, also sampled in North Shanxi, is genetically related and clustered close to a race 4 population sampled in the north of Shanxi Province. This suggests that X12 may have evolved based on a race 4 population, in contrast to LY1, which was artificially developed from race 2 and race 5 populations. SCN distribution and population density are relatively high in Shanxi (Lian et al., 2016) but commercial soybean production is lower in North Shanxi than in the more developed South Shanxi and the other main soybean production areas. This reduces the likelihood of the selection of complex SCN races by growing resistant cultivars at high production intensity. To substantiate our assumption, the location Gujiao, where we sampled X12 and race 4 SCN populations, was examined more closely. According to Mr. G. M. Gong (Gujiao city agricultural administration, personal communication, October 12, 2021), local people are growing predominantly two cultivars in Gujiao city for commercial use. We tested these two cultivars with KASP markers as mentioned before (Shi et al., 2015), and we found that both cultivars did not show the most common SCN resistance loci. Besides commercial production of soybean cultivars, Mr. Gong indicated that the local people are also growing on approximately 20% of the soybean growing area of a black seed coat, kidney-shaped landrace named Shenxingheidou in Gujiao city. A KASP marker analysis (Shi et al., 2015) revealed that this landrace is a mixture of Peking (45%) and PI 88788 (55%) resistance haplotypes. SCN of race 4 and X12 can reproduce on both resistance haplotypes and may have evolved under the selection pressure of this landrace. The landrace was heavily infested with SCN in the field (Lian, unpublished.). The same could possibly have happened anywhere in North Shanxi where SCN race 4 populations were found. Further research is needed to confirm this preliminary assumption, but this information indicates that complex virulence such as race 4 can evolve even under low selection pressure as seen in Gujiao city area.

Group 1.1 revealed the longest genetic distances to all other groups (ML tree), and in pairwise comparisons of the groups, the highest average *F*_ST_ values (Supplementary Table 3) and more frequent higher *F*_ST_ values of the single genomic sites (*F*_ST_ violin plot, Supplementary Figure 1). There are some genome areas with distinct *F*_ST_ peaks (Manhatten plot, Supplementary Figure 2), suggesting that there is a selection acting on some haplotypes depending on the geographic origin of the samples possibly as a result of the required adaption to the environment, but also may be depending on required virulence, e.g., there are several *F*_ST_ peaks for the comparisons of Group 1.1 (race 4) vs. others (other races) (Group 1.1 vs. Group 2, Group 1.1 vs. Group 1.2, Manhattan plot, Supplementary Figure 2).

## Discussion

Soybean plays an important role in agricultural production. It is not only the source of both vegetable protein and edible oil for human food, it is also the source of feed for animal and industrial processes (Hannah Ritchie and Roser, 2021). Facing the gap between limited domestic production of soybean and an expanding demand on one side and on the other side the continuous evolution of SCN virulence genes, it is of vital importance for China to promote domestic soybean production but also to reduce yield losses due to SCN damage (Yan and Baidoo, 2018; Peng et al., 2021). Additionally, this situation is aggravated by the fact that minor but significant losses due to SCN damage are easy to ignore by farmers (Niblack et al., 2008) but may be common in the main production areas in China according to the survey data (Lian et al., 2016; Chen et al., 2021) as summarized in Figure 1, and accumulated yield losses are relevant nation-wide. Wrather et al. (2001) estimated yield losses due to SCN damage of 5.5% for the production year 1998 in China. They estimated that SCN-specific losses may exceed 50% but only in some fields leaving a minimum of 10% of the acreage significantly damaged by the SCN. Consequently, the total affected acreage must be much larger. Newer data are not available but according to the increased occurrence of the SCN in China, the corresponding damage of the SCN may have increased over the past two decades.

Although the SCN has spread almost everywhere in the two major soybean planting areas in China, the planting area of cultivars with SCN resistance is very limited. So far, some varieties with SCN resistance have been registered already a long time ago in China but with almost no growing area, such as Zhonghuang 12 and Zhonghuang 57, a series of Kangxian cultivars (Liu et al., 2008; Duan and Peng, 2014; Peng et al., 2021). According to the survey of cultivars/breeding lines for use in the Huang-Huai Valleys, only a few of these exhibit SCN resistance loci (Lian et al., 2019a). Furthermore, of the cultivars with the top three acreages in each of the two major soybean planting areas in 2019, only one variety, named “Heinong 84,” exhibits SCN resistance, and the planting area accounts for only 4% of the soybean planting area in Heilongjiang. One of the reasons why cultivars with SCN resistance were planted in such a limited area may be that soybean yield reduction due to SCN damage occurs to some extent even in the absence of symptoms (Niblack et al., 2008). There is also no regular survey or service system available in China where the farmers can send soil samples for SCN testing as compared with the United States (Tylka and Marett, 2021). Therefore, farmers may not know that their fields are infected by SCN and that they are already suffering minor but significant yield losses due to SCN damage. Second, the yield of SCN-resistant cultivars is still lower compared with the popular local cultivars. SCN resistance breeding in China is still at an early developmental stage.

To avoid or at least to reduce the losses caused by SCN damage, we proposed (1) to implement periodic SCN monitoring including egg counting and race identification in the major soybean planting areas, (2) to promote and support wider crop rotations by the local government, and (3) to intensify funding on research programs and breeding programs related to SCN resistance to improve the breeding of broad SCN resistant, high yielding soybean cultivars. The inclusion of the SCN resistance in the cultivar registration system would provide transparency and increase awareness of this partially neglected threat. The key requirements for successful management of SCN are the acknowledgment and awareness of the growers and breeders that the SCN is an increasing threat to the sustainable development of the soybean production in China, and that the continuation of traditional practices will carry on driving SCN virulence evolution and accumulation, threatening the soybean production finally.

## Data Availability Statement

The original contributions presented in the study are included in the article/Supplementary Material, further inquiries can be directed to the corresponding author/s.

## Author Contributions

YL and WL designed the experiments. YL produced the data. JW, CL, and DB performed the bioinformatics analysis. YL drafted the manuscript with contributions from GK, HN, and WL. All authors read and approved the submitted version.

## Conflict of Interest

The authors declare that the research was conducted in the absence of any commercial or financial relationships that could be construed as a potential conflict of interest.

## Publisher’s Note

All claims expressed in this article are solely those of the authors and do not necessarily represent those of their affiliated organizations, or those of the publisher, the editors and the reviewers. Any product that may be evaluated in this article, or claim that may be made by its manufacturer, is not guaranteed or endorsed by the publisher.

## Footnotes

1
https://en.wikipedia.org/wiki/North_China_Plain

2
https://en.wikipedia.org/wiki/2021_East_Asia_sandstorm
