# Supplementary material for: The Spatial Distribution and Genetic Diversity of the Soybean Cyst Nematode, Heterodera glycines, in China: It Is Time to Take Measures to Control Soybean Cyst Nematode
Source: Front Plant Sci. 2022 Jun 15;13:927773. doi: 10.3389/fpls.2022.927773 (PMC9242501; doi:10.3389/fpls.2022.927773)
Supplement: Supplementary file 1 [file Data_Sheet_1.docx]

**SUPPLEMENTAL INFORMATION**

**SUPPLEMENTAL MATERIALS AND METHODS**

**Soybean cyst nematode population materials**

A total of 54 samples of *Heterodera glycines (H. glycines)*, including race1-6, race 11 and X12 population, collected from the provinces of Henan, Anhui, Shandong, Shanxi, Hebei, Jiangsu in China, were used in this study (Supplemental Table 1). All of the samples come from the survey for the presence of soybean cyst nematode, *H. glycines*, in the Huang-Huai Valleys during 2012-2015(Lian et al., 2016).

**Plant materials**

Seeds of Lee68 (susceptible check), ZDD2315, and PI 567516C were taken from the germplasm resources at the Henan Academy of Agricultural Science, Zhengzhou, China.

**Re-sequencing**

The raw paired-end reads were trimmed for adapters and low quality bases using Fastp v0.23.2(Chen et al., 2018). Cleaned read pairs were mapped to the *H. glycines* X12 genome (Lian et al., 2019) using BWA mem v0.7.17-r1188(Li, 2013), with –M option to mark split alignments as secondary. Alignments with mapping quality ≥ 20 were retained and duplicated reads were marked using Picard v 2.10.6 (<http://broadinstitute.github.io/picard/>). Variants were pre-called using [SAMtools](https://www.sciencedirect.com/topics/neuroscience/samtools) v1.13(Li et al., 2009) with recommended commands: “samtools mpileup -uf genome_file bam_file | bcftools call -Ov -mv.” Variants called by the two programs were filtered separately using the criteria: ⑴ variant quality ≥ 30; ⑵ depth ≥ 10 & ≤ 300; ⑶ no significant strand bias; and ⑷ no missing genotypes. The filtered sites were used for base quality score recalibration in the final variant calling using GATK HaplotypeCaller(Poplin et al., 2018). The GATK analysis was performed following the online Best Practices protocol with default parameters (<https://software.broadinstitute.org/gatk/best-practices/>). Variants called by GATK were filtered using bcftools (<http://samtools.github.io/bcftools/>) with the criteria: 1) variant quality ≥ 30; 2) minimal depth for each sample ≥ 2; 3) at least 20 bp away from an [InDel](https://www.sciencedirect.com/topics/biochemistry-genetics-and-molecular-biology/indel); and 4) no missing genotypes. The resulting genome size was 141,354,287 bp and the population sample comparison rate was between 74.59% and 93.64%.

**Phylogenic analyses**

Maximum-likelihood phylogenetic tree was generated using IQ-TREE v2.1.4-beta(Minh et al., 2020). Best nucleotide substitution model was chosen by model test function in IQ-TREE, and ascertainment bias correction (ASC) was applied for likelihood calculation on SNP data. Finally, the Variable Time (VT+F+ASC+R4) model were used on phylogeny inference for SNPs. The program was run with 1000 bootstrap replicates and *Globodera rostochiensis*(Eves-van den Akker et al., 2016), Gr2016, is used as outgroup species.

**Analyses of genetic structure among populations**

To estimate the population genetic differentiation, unbiased estimates of F_ST_ based on Hudson et al. (1992) for population pairs were calculated using VCFtools to evaluate F_ST_ based on 10 kb sliding windows in 2 kb steps(Petr et al., 2011).

**Bioassay**

ZDD 2315 and PI 567516C were evaluated for X12 population infestation following an environmental chamber bioassay at the Henan Academy of Agricultural Science, Zhengzhou, China. Briefly, plastic cups (6 cm×12 cm) were filled with soil infected by X12 population. The susceptible cultivar Lee68 was planted in several cups rearing a sufficient number of eggs for the following bioassay. Six plants of each ZDD 2315, PI 567516C and Lee68 were transplanted in individual cups and the cups arranged in a randomized complete block design. Three days after transplantation, seedlings were inoculated with each about 2,500 eggs of X12. The experiments were maintained at a relative humidity of 70-80% and the temperature was set to 28℃ during the light photoperiod of 16 hours and 25°C during night. Plants were watered daily. Thirty days post inoculation, nematode cysts were washed from the roots of each plant and nematode cysts images were captured with a digital camera. The number of cysts were counted from the image.

**Supplemental figures 1-2**


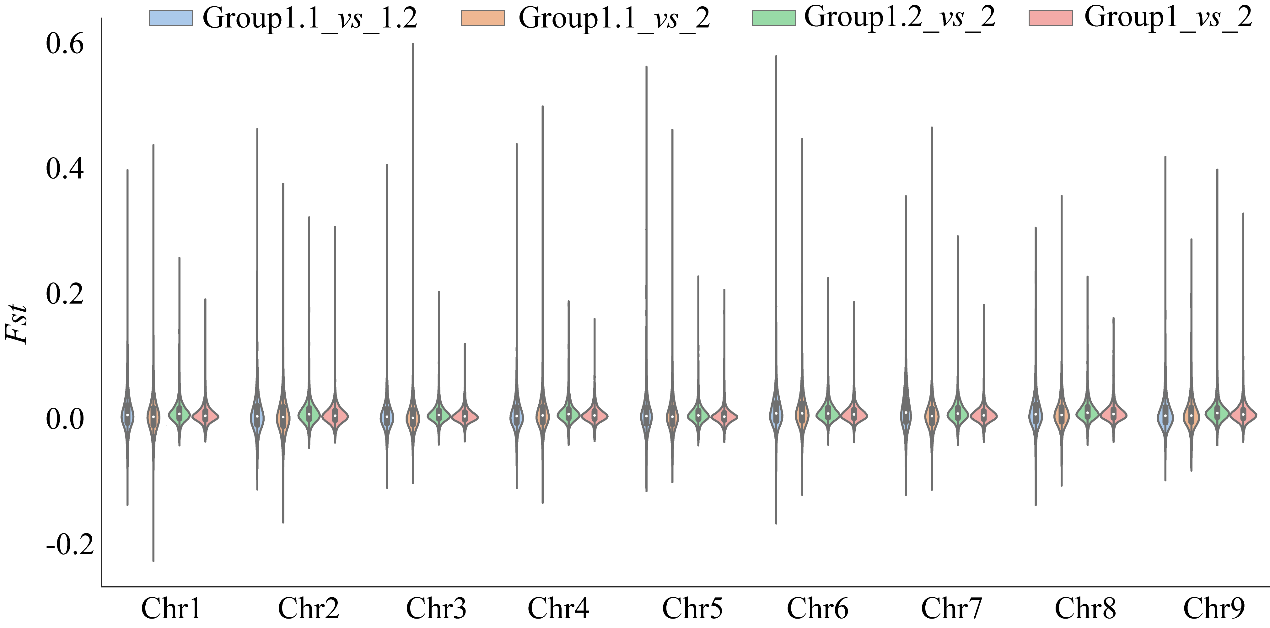


**Figure 1.** Violin plot of genome-wide F_ST_ values between selected comparisons of groups according the phylogenetic tree of SCN populations in the Huang-Huai Valleys in China.


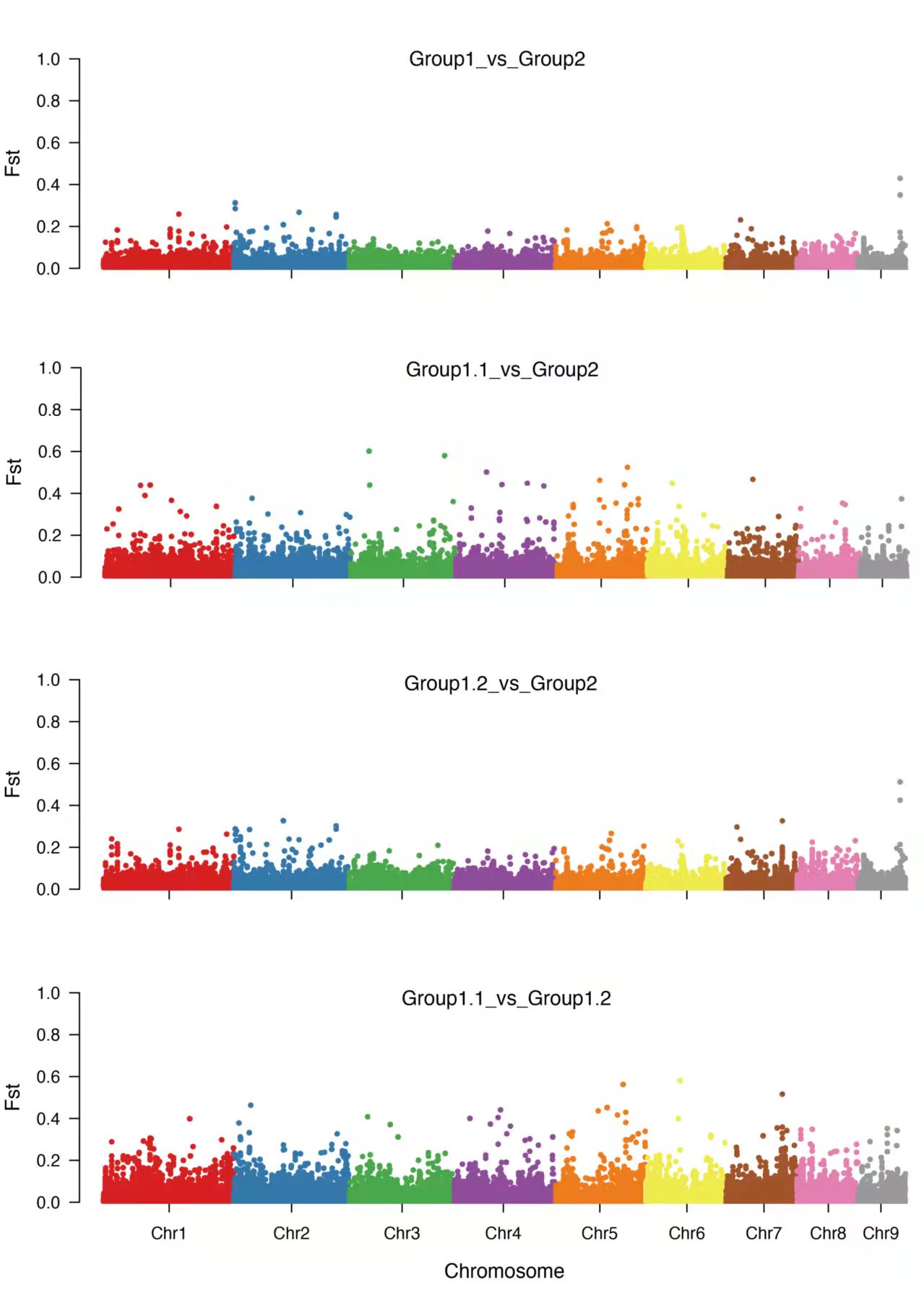


**Figure 2.** Manhattan plot of genome-wide F_ST_ values between selected comparisons of groups according the phylogenetic tree of SCN populations in the Huang-Huai Valleys in China.

**References:**

Chen, S., Zhou, Y., Chen, Y., and Gu, J. (2018). fastp : an ultra-fast all-in-one FASTQ preprocessor. Bioinformatics, 34: i884-i890.

Eves-van den Akker, S., Laetsch, D. R., Thorpe, P., Lilley, C. J., Danchin, E. G., Da Rocha, M., et al. (2016). The genome of the yellow potato cyst nematode, Globodera rostochiensis, reveals insights into the basis of parasitism and virulence. Genome Biology, 17: 124.

Hudson, R. R., Slatkin, M., and Maddison, W. P. (1992). Estimation of levels of gene flow from DNA sequence data. Genetics, 132: 583-589.

Li, H. (2013). Aligning sequence reads, clone sequences and assembly contigs with BWA-MEM. Genomics.

Li, H., Handsaker, B., Wysoker, A., Fennell, T., Ruan, J., Homer, N., et al. (2009). The sequence alignment/map format and SAMtools. Bioinformatics, 25: 2078-2079.

Lian, Y., Wang, J. S., Li, H. C., Wei, H., Li, J. Y., Wu, Y. K., et al. (2016). Race distribution of soybean cyst nematode in the main soybean producing area of Huang-Huai rivers valley. Acta Agronomica Sinica, 42: 1479-1486.

Lian, Y., Wei, H., Wang, J. S., Lei, C. F., Li, H. C., Li, J. Y., et al. (2019). Chromosome‐level reference genome of X12, a highly virulent race of the soybean cyst nematode *Heterodera glycines*. Molecular Ecology Resources, 19: 1637-1646.

Minh, B. Q., Schmidt, H. A., Chernomor, O., Schrempf, D., and Lanfear, R. (2020). IQ-TREE 2: New Models and Efficient Methods for Phylogenetic Inference in the Genomic Era. Molecular biology and evolution, 37: 1530-1534.

Petr, Danecek, Adam, Auton, Goncalo, Abecasis, et al. (2011). The variant call format and VCFtools. Bioinformatics, 27: 2156-2158.

Poplin, R., Ruano-Rubio, V., DePristo, M. A., Fennell, T. J., Carneiro, M. O., Van der Auwera, G. A., et al. (2018). Scaling accurate genetic variant discovery to tens of thousands of samples. bioRxiv.
